# Expanding the Neurological Phenotype of Ring Chromosome 10 Syndrome: A Case Report and Review of the Literature

**DOI:** 10.3390/genes12101513

**Published:** 2021-09-26

**Authors:** Jacopo Pruccoli, Claudio Graziano, Chiara Locatelli, Lucia Maltoni, Hodman Ahmed Sheikh Maye, Duccio Maria Cordelli

**Affiliations:** 1 Child Neurology and Psychiatry Unit, IRCCS Istituto delle Scienze Neurologiche di Bologna, 40138 Bologna, Italy; jacopo.pruccoli@studio.unibo.it; 2 Dipartimento di Scienze Mediche E Chirurgiche (DIMEC), University of Bologna, 40138 Bologna, Italy; 3 UO Genetica Medica, IRCCS Azienda Ospedaliero-Universitaria di Bologna, 40138 Bologna, Italy; claudio.graziano@unibo.it; 4 Neonatal Intensive Care Unit, S.Orsola Malpighi Hospital, 40138 Bologna, Italy; chiara.locatelli@aosp.bo.it; 5 Child Neurology and Psychiatry Unit, Azienda USL della Romagna, 48121 Ravenna, Italy; lucia.maltoni3@unibo.it; 6 IRCCS Istituto Delle Scienze Neurologiche di Bologna, UOC Neuroradiologia, 40139 Bologna, Italy; hodman.ahmedsheikhmaye@isnb.it

**Keywords:** ring chromosome 10, r10, r(10), neurology, neuroradiology, magnetic resonance imaging (MRI), *ZMYND11*, *EBF3*

## Abstract

Ring chromosome 10 [r(10)] syndrome is a rare genetic condition, currently described in the medical literature in a small number of case report studies. Typical clinical features include microcephaly, short stature, facial dysmorphisms, ophthalmologic abnormalities and genitourinary malformations. We report a novel case of r(10) syndrome and review the neurological and neuroradiological phenotypes of the previously described cases. Our patient, a 3 year old Italian girl, represents the 20th case of r(10) syndrome described to date. Intellectual disability/developmental delay (ID/DD), microcephaly, strabismus, hypotonia, stereotyped/aggressive behaviors and electroencephalographic abnormalities were identified in our patient, and in a series of previous cases. A brain MRI disclosed a complex malformation involving both the vermis and cerebellar hemispheres; in the literature, posterior cranial fossa abnormalities were documented by CT scan in another case. Two genes deleted in our case (*ZMYND11* in 10p and *EBF3* in 10q) are involved in autosomal dominant neurodevelopmental disorders, characterized by different expressions of brain and posterior cranial fossa abnormalities, ID/DD, hypotonia and behavioral problems. Our case expands the neurological and neuroradiological phenotype of r(10) syndrome. Although r(10) syndrome represents an extremely rare condition, with a clinical characterization limited to case reports, the recurrence of specific neurological and neuroradiological features suggests the need for specific genotype-phenotype studies.

## 1. Introduction

Ring chromosome 10 [r(10)] syndrome represents a rare genetic condition, resulting from de novo breakage of chromosome 10 and subsequent intrachromosome fusion during meiosis or initial postzygotic mitosis. It is currently reported in 19 unrelated patients in the medical literature, but most patients were described before the introduction of chromosomal microarray technology, and the precise definition of deleted regions and encompassed genes is lacking. Typical clinical features include intellectual disability and/or developmental delay (ID/DD), microcephaly, short stature, facial dysmorphisms, ophthalmologic abnormalities and genitourinary malformations [1,2,3,4,5,6,7,8,9,10,11,12,13,14,15,16,17]. In constitutional ring chromosomes, the regions involved by the breakage, the amount of lost information, and the level of mosaicism resulting from the unstable nature of the ring upon cell division may vary each time, causing patients with a seemingly analogous ring chromosome to display diverse clinical pictures [18]. We report the case of a novel patient with r(10) syndrome, presenting a review of previously described neurological and neuroradiological phenotypes.

## 2. Case Presentation

Our patient is a 3 year, 10 month old girl born to non-consanguineous, Italian parents. Family history revealed a congenital ventricular septal defect in the mother.

A first trimester combined prenatal screening (assessing serum pregnancy-associated plasma protein-A (PAPP-A) and human chorionic gonadotropin (HCG), and ultrasound evidence of nuchal translucency) was unremarkable. Intrauterine growth retardation was observed at 28 weeks of gestational age. No exposure to radiation or any drug was registered. She was born at 35 weeks, 4 days of gestational age by caesarean section for podalic version. At birth, the patient presented diffused hypotonia with impaired suction, bilateral pes valgus and a systolic heart murmur. Weight at birth was 1.610 g (−2 SD); length 42 cm (−2 SD); and head circumference 29 cm (−2 SD) [19]. APGAR scores were 5 at 1′, and 9 at 5′. At birth, she was hospitalized for 20 days in a Neonatal Intensive Care Unit for respiratory distress, neonatal jaundice and hypocalcemia. Cerebral ultrasounds revealed ventricular asymmetry (left prominence). Cardiological examination showed ostium secundum atrial septal defect, patent ductus arteriosus and left ventricular dilation with moderate systolic dysfunction, requiring treatment with furosemide and captopril. Given the evidence of recurrent aspiration pneumonias occurring since her infancy, multiple chest radiographs were performed, which did not reveal potentially responsible malformations in the upper or lower airways. Repeated upper gastrointestinal tract radiographies were then performed, documenting multiple episodes of gastroesophageal reflux, followed by aspiration into the upper airways. The cough reflex was unremarkable, and no malformation of upper or lower gastrointestinal tract was documented. Neonatal otoacoustic emissions were unremarkable.

Cytogenetic analysis of blood lymphocytes revealed an apparently non-mosaic ring chromosome 10 [46,XX,r(10) (p15q26.1)]. Fifty metaphase spreads were analyzed in each experiment to exclude mosaicism. FISH analysis with chromosome 10 subtelomeric probes revealed secondary aberrations in 40% of the examined mitoses (mainly a ring chromosome 10 with conserved subtelomeric regions and a derivative chromosome 10 with two subtelomeric p and one q region on the short arm). Chromosomal microarray analysis (SurePrint G3 ISCA v2 CGH 8x60K, Agilent Technologies) allowed a better definition of the breakpoints: arr[GRCh37] 10p15.3p15.1(136361_4212930) × 1,10q26.13q26.3(127294919_135434178) × 1, indicating an approximately 4.1 Mb deletion in 10p and 8.2 Mb deletion in 10q. These deletions encompass many protein-coding genes, but we highlight that two deleted genes (*ZMYND11* in 10p and *EBF3* in 10q) are involved in autosomal dominant neurodevelopmental disorders, with haploinsufficiency as the accepted pathogenic mechanism. We thus believe that *ZMYND11* and *EBF3* are likely the major drivers of the neurological phenotype in this patient. The full list of the genes encompassed by the deletion documented in our patient are reported in Table 1, together with their discussed functions and phenotypes [20,21,22].

**Figure 1:**
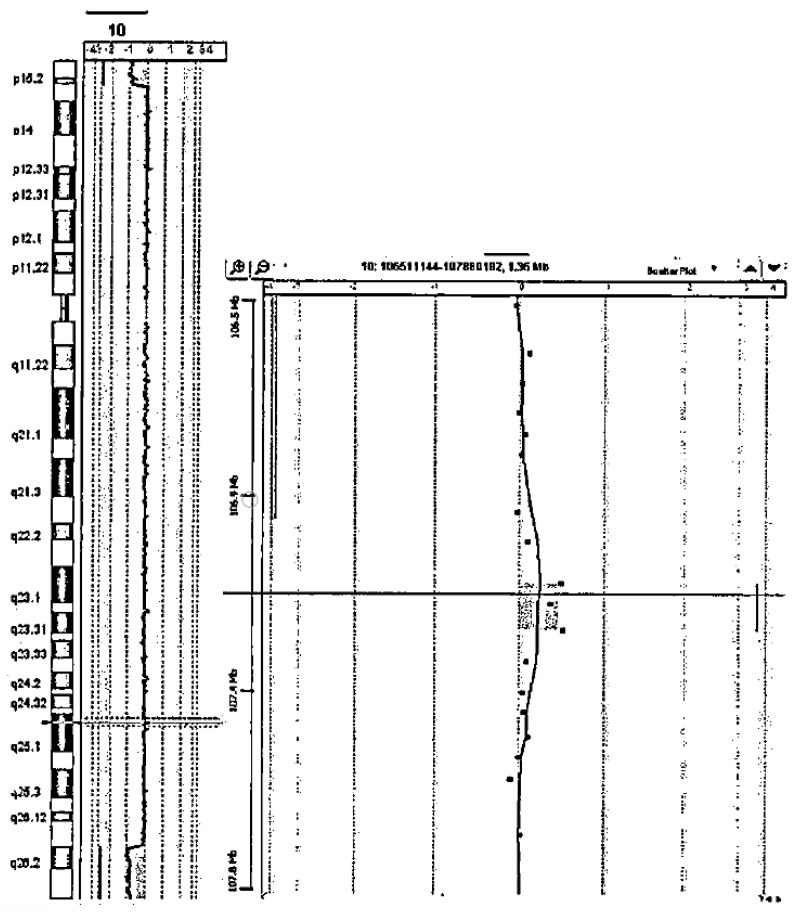
Microarray profile of the present case.

Parental analysis was not performed, in accordance with the caregivers of the patient. The microarray profile for this patient is reported in Figure 1.

For markedly reduced growth rates, and growth hormone (GH) deficiency, GH treatment was started at the age of 3 years. At 3 years, 2 months, before the start of GH, body weight was 9.0 kg (<2.5 SD), height was 77.8 cm (<2.5 SD), and head circumference was 42 cm (<2.5 SD). Her growth improved after 8 months of GH therapy, reaching a body weight of 12.5 kg (<2 SD) and height of 87 cm (<2.5 SD). Lumbar kyphosis, abduction and external rotation of the hips, and pes valgus, required the adoption of individualized postural systems. Ophthalmological evaluations revealed myopia and convergent strabismus.

Repeated neurological evaluations of our patient showed severe intellectual disability and global developmental delay. Head control was gained at 6 months. At 12 months of life, she reached an autonomous sitting position. At around 3 years she started to keep upright position autonomously. At 3 years, 10 months, our patient maintains an upright position and walks if supported. Spoken language is absent and the declarative pointing gestures have not been acquired. Augmentative and alternative communication was recently started. She frequently presents body rocking stereotypies; acute, paroxysmal hair-pulling stereotypies are occasionally evident. Significant traction alopecia occurred as a consequence of these stereotypies, requiring hair cutting. Divergent, congenital strabismus persisted since the first months of life with alternating esophoria. Remaining cranial nerves are clinically unremarkable. Micrognathia is evident. She presents axial hypotonia and deep tendon reflexes are bilaterally brisk. She uses both hands to manipulate and play with objects, showing good interactions with caregivers and examiners. A sleep and wake EEG performed at 1 year, 4 months, revealed diffuse rapid rhythms, prevalent on temporo-parietal regions of both hemispheres. The background activity showed posterior bilateral slowing, more evident on the parieto-occipital region of the right hemisphere and at the vertex. These elements persisted at 3 years. No defined paroxysmal activity was identified, and our patient has never presented any febrile or non-febrile seizures.

**Figure 2:**
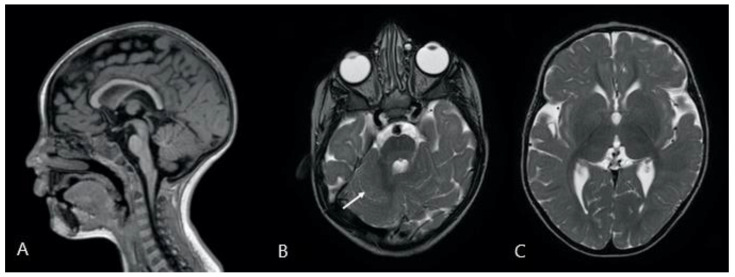
Description: (**A**): 3D-T1-weighted midline sagittal image shows a reduced thickness of the corpus callosum; the pons appears to be small and short compared to the length of the midbrain and medulla oblongata. The vermis is slightly hypoplastic with a prevalent reduction in height. (**B**,**C**): Turbo Spin Echo T2-weighted axial images showing (**B**) dysplastic cerebellum with abnormal foliation (white arrow), and (**C**) dysmorphic lateral ventricles, especially the frontal and occipital horns.

Two brain magnetic resonance imaging (MRI) studies, performed on a 1.5T scanner (Achieva, Philips) when the patient was 2 years, and 3 years and 10 months old, respectively, revealed the presence of several abnormalities, mostly involving structures of the posterior cranial fossa. The cerebellum appeared dysplastic with abnormal foliation, especially the anterior lobe, and a mild asymmetry of the hemispheres was present. The vermis was mildly hypoplastic, with a prevalent reduction in height rather than anteroposterior diameter [23]. Pontine hypoplasia with abnormally increased rostrocaudal length of the medulla and midbrain with respect to the pons was present. Corpus callosum was hypoplastic with reduced thickness of the rostrum and splenium, whose measurements were below the 3rd percentile, and the genu and isthmus thickness were reduced compared to the median values [24]. Lateral ventricles were enlarged and dysmorphic, presenting wavy margins and a widened aspect of the frontal horns. Periventricular white matter appeared reduced in volume. The main findings of the brain MRI are reported in Figure 2.

## 3. Search Strategy and Analysis of Literature

Citations were identified through PubMed, Web of Science and Google scholar searches using the search terms (including variations), ‘‘Ring chromosome 10 syndrome”, ‘‘Ring 10 neurology”, “Ring 10 neuroradiology” and “Ring 10 electroencephalography”, combined with study filters for original research, case reports and case series. The identified reports were screened manually for patients fulfilling inclusion criteria (Ring chromosome 10 syndrome). Additional articles were identified from the reference lists of identified papers. Only papers published in English were reviewed. Nineteen previously published cases were identified in the literature [1,2,3,4,5,6,7,8,9,10,11,12,13,14,15,16,17].

## 4. Results

Together with the presented case, 20 cases were identified. The main findings are summarized in Table 2. Fifteen subjects presented ID/DD. Microcephaly was reported in 15, and eight patients presented strabismus. Hypotonia was identified in nine patients. An EEG description was provided in six cases, of which one patient presented EEG abnormalities. One patient had a history of seizures time linked to progressive renal failure and three patients presented febrile seizures. Five patients were studied with a brain computerized tomography (CT), whereas in 2/20 cases a brain MRI was performed. In one case, a CT scan documented a Dandy–Walker variant.

## 5. Discussion

Ring 10 chromosome syndrome is an uncommon disease, with 19 previously reported cases in literature. Clinical neurological and neuroradiological features presented in our patient partially resemble those documented in a small series of previously documented cases.

Clinically, our patient presents ID/DD. ID and DD, from mild to severe, have been reported in nearly all described patients with r(10) syndrome surviving across childhood. As an exception, case n° 17 presented normal development with no reported ID but died at age 15 years due to cardiac arrest [16].

Since her birth, our patient presented microcephaly. Microcephaly is a relatively stable characteristic across r(10) cases with different genetic involvement, as it is reported among 15/20 patients. Notably, case n° 16, an autopsy study of a 27 gestational week old fetus, represents the only case of r(10)-associated macrocephaly [15]. Concerning ophthalmological involvement, our patient presents convergent strabismus and myopia, two conditions previously reported in patients with r(10) syndrome (8/20 and 4/20, respectively). Possible coexistence of choroid coloboma, macular hypoplasia or Horner Syndrome confirm the need to perform an ophthalmological screening in these patients. Truncal hypotonia, as revealed by neurological examination of our patient, is frequently the most evident clinical neurological finding among patients with r(10) syndrome. Hypotonia, sometimes associated with reduced muscle mass, has been reported among 9/20 cases, and may range from mild to severe. No other major specific focal neurologic deficit has been described to date. Nonetheless, our patient presents body rocking- and severe hair picking-like stereotypes; remarkably, case n° 19 presented stereotyped repetitive hand movements, pressing her palms in the midline, and stroking her thumbs [17]. Concerning psychopathological and psychiatric comorbidities, hyperactivity and mood disorders have been sporadically associated.

In our patient, EEGs showed diffuse rapid rhythms prevalent on temporo-parietal regions of both sides, and posterior slowing. No febrile or non-febrile seizures were documented by history taking or EEGs. EEG and epileptological aspects of r(10) syndrome have not been studied extensively to date. Febrile seizures have been reported in 3/20 cases, with a normal EEG in two cases. None of those patients developed epilepsy. Non-febrile, treatment-resistant seizures have been described in one case at 40 days of life; seizures were time-linked to progressive renal failure, which led the patient to death during his third month of life. No associated EEG finding was reported [12]. EEG abnormalities were described only in one patient (case n° 6), reported as the presence of slow waves in occipital regions, associated with high voltage, diffuse fast activity and paroxysmal generalized slow waves [6]. Seizures were not reported for this patient.

Brain MRI disclosed in our patient dysplastic cerebellum with hypoplastic vermis, pontine hypoplasia, hypoplastic corpus callosum and lateral brain ventricle abnormalities. As for previously described patients, one was studied with a brain MRI and five with a brain CT scan. The brain MRI and 4/5 brain CTs were normal, whereas case n° 18 presented a posterior fossa malformation described as Dandy–Walker variant [14]. Dandy–Walker variant is a rare congenital intracranial malformation, comprising the spectrum of abnormalities of the posterior fossa, characterized by cystic posterior fossa mass and variable hypoplasia of the cerebellar vermis, without enlargement of the posterior fossa [25]. To the best of our knowledge, our patient represents the first report of neuroradiological MRI findings in an r(10) chromosome syndrome. The multiple and complex brain abnormalities detected using the MR scans are probably the consequence of loss of protein-coding genes involved in the local proliferation, migration and/or differentiation of the nervous tissue, concerning in particular the structures located in the posterior cranial fossa.

Our patient presents an approximately 4.1 Mb deletion in 10p and 8.2 Mb deletion in 10q, affecting several protein-coding genes. A number of these genes have been previously associated with DD/ID, such as *ZMYND11*, *EBF3*, *DIP2C*, *PRR26*, and *IDI2* [16,17].

*ZMYND11* (Zinc finger MYND domain containing protein 11), encompassed by the 10p15 deletion in our patient, acts as a transcriptional repressor. Haploinsufficiency of *ZMYND11* is believed to play the main role in chromosome 10p15.3 microdeletion syndrome, a condition characterized by ID/DD, particularly affecting speech, craniofacial dysmorphism, hypotonia and seizures [26]. An association with behavioral abnormalities has been frequently reported, including aggressive behaviors, attention deficit, hyperactivity, and impulsivity and autistic traits [27]. Documented brain MRI abnormalities in these patients include cortical atrophy, pachygyria, periventricular leukomalacia and delayed myelination [28].

*EBF3* (Early B Cell Factor 3), a gene involved in the deletion identified in our patient in 10q26, has a possible role in neuronal differentiation and maturation. A series of interstitial deletions affecting many genes and including *EBF3* has been described in patients with ID/DD. More specifically, *EBF3* point mutations were reported to cause a condition named Hypotonia, Ataxia, and Delayed Development Syndrome (HADDS). A small number of patients with HADDS present cerebellar vermian hypoplasia [29]. Notably, a recent report describes a patient with a small deletion affecting almost exclusively *EBF3*, clinically overlapping patients with larger deletions [30].

These considerations indicate that haploinsufficiency of *ZMYND11* and *EBF3* are likely the major drivers in determining the r(10) syndrome phenotype in this patient with a complex neurological involvement, characterized by ID, DD, hypotonia, behavioral abnormalities and MRI anomalies.

## 6. Conclusions

This report represents the first description of brain MRI alterations in a patient with r(10) syndrome. The neurological and neuroradiological findings here reported expand the clinical spectrum of r(10) syndrome and are likely associated with haploinsufficiency of *ZMYND11* and/or *EBF3*. These findings provide further support of genotype-phenotype correlations in r(10) syndrome, and further descriptions of patients with this condition may help the recognition, management and definition of their global clinical spectrum.

## Figures and Tables

**Table 1:** Genes interested by deletions identified in the present case.

Deletion	Gene ^a^	Gene OMIM Number	Transmission	Function	Phenotype and Phenotype OMIM Number	Discussion
1	*ZMYND11*	608668	AD	Zinc finger	OMIM 616083 Mental retardation, autosomal dominant 30	ID/DD and behavioral problems documented in the present case; differences in MRI abnormalities
1	*WDR37*	618586	AD	Protein involved in cell cycle, transduction and apoptosis	OMIM 618652 Neuro-oculo-cardio-genito-urinary syndrome	ID/DD with prevalent language impairment, hypotonia, microcrania and CNS abnormailities partially overlap the present case major heart and eye abnormalities
1	*PITRM1*	618211	AR	Metallopeptidase	OMIM 619405 Spinocerebellar ataxia, autosomal recessive 30	DD +/− ID, ataxia, behavioral abnormalities. Cerebellar atrophy. Progressive condition.
2	*MMP21*	608416	AR	Matrix metalloproteinase	OMIM 616749 Autosomal visceral heterotaxy-7	Major cardiac abnormalitites (minor cardiac abnormalities in the present case). Situs inversus
2	*EBF3*	607407	AD	Trasciption factor inducing apoptosis and cell cycle arrest	OMIM 617330 Hypotonia, ataxia, and delayed development syndrome	Hypotonia, ID/DD, ataxia; in a few cases, cerebellar vermian hypoplasia, partially overlapping the present case
2	*ECHS1*	602292	AR	short-chain enoyl-CoA hydratase, catalyzing mitochondrial fatty acid β-oxidation	OMIM 616277 Mitochondrial short-chain enoyl-CoA hydratase-1 deficiency	DD, hyptonia, brain atrophy and brain lesions in the basal ganglia. Progressive condition

Deletion 1: del(10)(p15.3p15.1); deletion 2: del(10)(q26.13q26.3). Abbreviations: AD: autosomal dominant; AR: autosomal recessive; CNS: central nervous system; DD: developmental delay; ID: intellectual disability; MRI: magnetic resonance imaging. ^a^: Online Mendelian Inheritance in Man (OMIM) (https://www.omim.org) (accessed on 19 September 2021) [20]; DECIPHER (https://www.deciphergenomics.org (accessed on 19 September 2021)) [21]; GeneCards (https://www.genecards.org (accessed on 19 September 2021)) [22].

**Table 2:** Neurological phenotype of described patients with ring 10 chromosome syndrome.

Case	Breakpoints (10p and 10q)	ID/DD	Microcephaly	Strabismus	Hypotonia	Other	EEG	Seizures	Brain Imaging	Reference
1	p14, q25	+	+							Lansky et al. (1977)
2	p15, q26	+	+	+		Hyperactivity				Sparkes et al. (1978)
3	p15, q25		+		+				Normal CT	Fryns et al. (1978)
4	p15, q26	+	+		+					Simoni et al. (1979)
5	p15, q26	+	+	+			Normal	FS	Normal US	Tsukino et al. (1980)
6	p15.3, q26.1	+	+		+		Occipital SW; diffuse fast activity; paroxysmal generalized SW		Normal CT	Michels et al. (1981)
7	p15, q26	+	+							Serville et al. (1982)
8	p15, q26	+	+				Normal	FS		Nakai et al. (1983)
9	p15.3, q26.3	+		+			Normal		Normal CT	Kondo et al. (1984)
10					+					Zorn (1984)
11	p15, q26	+	+	+						Shapiro et al. (1985)
12	p15.3, q26.3	+					Normal			Kishi et al. (1985)
13	p13-15, q26	+	+		+			Convulsions		Calabrese et al. (1994)
14	p15, q26	+	+			MDD			Normal CT	Concolino et al. (2003)
15	p15.3, q26.12		+		+	Horner Syndrome			Normal MRI	Gunnarsson et al. (2009)
16	p14, q26.3		Macrocephaly							Christopoulou et al. (2012)
17	p15.3, q26.2		+	+						Guilherme et al. (2013)
18	p15.3, q26.13	+		+	+			FS	Dandy-Walker variant at CT	Guilherme et al. (2013)
19	p15.1, q26.1	+	+	+	+	Stereotypies			US: dilated ventricles, then normal	Čiuladaitė et al. (2015)
20	p15, q26.1	+	+	+	+	Stereotypies	Temporo-parietal BL rapid rhythms, posterior slowing	None	MRI: posterior cranial fossa and CC abnormalities	Present case

Abbreviations: BL: bilateral; CT: computerized tomography; CC: corpus callosum; DD: developmental delay; EEG: electroencephalography; FS: febrile seizures; ID: intellectual disability; MDD: major depressive disorder; MRI: magnetic resonance imaging; US: ultrasounds; SW: slow waves. Note: “+” indicates documented findings, blank spaces indicate missing data.

## Data Availability

The data that support the findings of this study are available from the corresponding author upon reasonable request.
